# Photogrammetric Analysis of Upper Cross Syndrome among Teachers and the Effects of National Academy of Sports Medicine Exercises with Ergonomic Intervention on the Syndrome

**Published:** 2019-07-03

**Authors:** Razieh Karimian, Nader Rahnama, Gholamali Ghasemi, Shahram Lenjannejadian

**Affiliations:** ^1^Department of Sports Injuries and Corrective Exercises, School of Sport Sciences, University of Isfahan, Isfahan, Iran

**Keywords:** UCS software, Forward head, Rounded shoulder, Hyperkyphosis

## Abstract

**Background:** Hyperkyphosis is often accompanied by forward head and shoulder postures. Together, these three disorders are called "Upper Cross Syndrome (UCS)". We aimed to perform a photogrammetric analysis of UCS among teachers and to determine the effects of National Academy of Sports Medicine (NASM) exercises with ergonomic training interventions on the syndrome.

**Study design:** A semi-experimental study.

**Methods:** Photogrammetric analysis was performed using the UCS software among teachers in order to determine the angles of forward head, rounded shoulders, and hyperkyphosis. Twenty-three teachers were selected purposefully and enrolled in Fasa City in 2018. They were randomly divided into experimental (n=12) and control (n=11) groups. Experimental group attended 12 wk of NASM exercises with ergonomic intervention but the control group did not participate in any regular exercise. The data were analyzed using paired t-test and differential independent t-test (*P*<0.05).

** Results:** The results indicated a significant decrease in forward head (*P*=0.001), shoulder angles (*P*=0.000) and hyperkyphosis (*P*=0.003). The applied intervention had a 90% positive effect in reducing the forward head angle, an 88% positive effect in reducing the rounded shoulder angle and a 90% positive effect in reducing the kyphosis angle. However, the results for the control group did not show a significant difference for forward head, rounded shoulders, and hyperkyphosis angles.

**Conclusion:** The UCS software application can be used as an accurate instrument for measuring the extent of the UCS. Moreover, using NASM exercises can lead to a reduction in the UCS among teachers.

## Introduction

Muscular imbalances can have certain consequences in the body^1^. Janda classifies these muscular imbalance patterns into three types including UCS, LCS, and layered syndrome. The UCS occurs in the neck and the scapulae areas. In this syndrome, upper posterior and anterior muscles in the neck, which are tonic muscles, are shortened and the deep posterior muscles of the spine in the neck area and the lower posterior muscles of the scapulae, which are mainly phasic muscles, are restrained, stretched, and weakened^2^. The prevalence of this abnormality is reported 11%-60% in different societies and ages^3^. A number of comprehensive studies are carried out on the effectiveness of corrective exercises and reduction of the abnormality. Comprehensive corrective exercise was effective on the forward head and forward shoulder angles, as well as spinal column curvature of patients with the UCS, and demonstrated that the mixed training program was much better than the other two programs^4^. Moreover, corrective exercises were effective on the UCS in patients with paraplegia spinal cord injury ^5^.

The design of exercise for preventing or reducing abnormalities have failed to consider the role of posture in repetitive movements of the work environment, neither have they focused on training of ergonomic principles. On the other hand, researches done in the workplace considered the effect of training interventions and/or corrective training on upper limb musculoskeletal disorders^6^. Therefore, musculoskeletal abnormalities especially, UCS were not studied in the work environment. Since the different repetitive and long-term situations in the workplace have always been mentioned as the main reasons in abnormalities of UCS^7^, one of the main topics, which is in the center of attention by many investigators, is believed to provide the suitable exercise and environment with ergonomic training for the organizations, institutions, and workers^8^. A number of therapeutic methods^4, 5, 9, 20^ have been introduced for patients with the UCS, which include physical therapy, body condition retraining, and using adhesive tapes, as well as auxiliary devices and exercises. Meanwhile, therapeutic exercises are recognized as a common method^9^.

NASM has recently proposed a new protocol for corrective exercises, which includes four stages of inhibiting, lengthen, activate, and integrate techniques. In this protocol, instead of solely lengthening the shortened or stiff muscle, it is better to first use inhibiting exercises, followed by lengthening exercises and activating and integrating exercises on the muscle. Therefore, since forward head, forward shoulder, and kyphosis abnormalities are closely related through a chain reaction^4, 10^. It does not seem that the separate correction of each one of them in an isolated manner has any scientific justification^4^ and since there has not yet been a study in this regard with a focus on teachers and using the UCS software, the main objective of the current study was to perform a photogrammetric analysis on UCS among teachers and to investigate the effects of the NASM exercises with ergonomic training intervention on this syndrome.

## Methods

In this semi-experimental study, statistical population included all the teachers currently teaching in schools around Fasa City located in south-west of Iran in 2018. After visiting the Education Organization and making the necessary arrangements, those teachers suffering from upper cross syndrome were selected as the sample in a targeted and convenient manner. Table 1 demonstrates that the participants of the study were 23 non-athletic teachers, divided into two groups including the NASM corrective exercise with ergonomic intervention and the control group after they completed the consent form (The Ethics Code, IR.UI.REC.1397.104).

**Table 1:** Subject characteristics

**Variables**	**Experimental (n=12)**		**Control (n=11)**	
	**Mean**	**SD**	**Mean**	**SD**
Age (yr)	45.2	8.1	44.1	7.8
Weight (kg)	78.3	13.1	77.9	13.0
Height (cm)	178.8	6.5	175.8	7.0

The criteria for entering the participants into the study included simultaneous suffering of three complications including hyperkyphosis of more than 42 degrees, forward head posture of more than 45 degrees, and rounded shoulder posture of more than 52 degrees^11^. The elimination criteria for the study included pathologic symptoms such as a history of surgery or fracture or joint diseases of the spine, osteoporosis, acute rheumatoid arthritis, blood diseases, congestive heart failure, malignancy, severe skin sensitization, and athletic activities.

In order to evaluate the angles of forward head and shoulder and kyphosis using photogrammetric method from the lateral view, we placed a number of landmarks on certain points. The C7 and T12 markers are highlighted to be clearly visible from the lateral view. In order to measure the angles of forward head and shoulders using this method, at first three anatomic markers of ear tragus, the acromion process, and the spinous process of the seventh vertebra of the neck were marked. Then, the participant posed in a profile manner and a digital camera was used to take a picture of his or her lateral side view from a distance of 265 centimeters^12^. Then, using the USC software application, the angle of the line connecting the tragus and the seventh neck vertebra with the perpendicular line (the forward head angle) as well as the angle between the acromion process and the perpendicular line (the rounded shoulder angle) were measured^12^. This method provides good reliability (the proposed method and the flexible ruler method have a 98% correlation), and it has been successfully used in various studies^11-14^. Moreover, in order to measure the angle of the kyphosis from the bone marker of the spinous process of the seventh vertebra was used as the starting point of the arc and the spinous process of the 12th dorsal vertebra, T12, was used as the endpoint of the arch^15, 16^. This method has a high correlation coefficient with the Cobb angle (0.906). Moreover, the sensitivity of 85% and the characteristic of 97% have been reported for it^16^.

**Figure 1:**
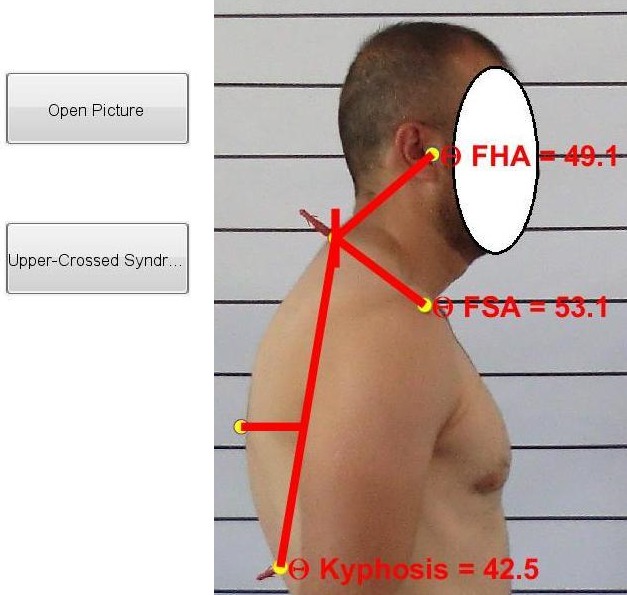
UCS software application

After determining the locations of the selected points, the participant is asked to stand in a natural and comfortable way with bare feet on a piece of cardboard with feet markers on it in a lateral manner and look forward while balancing his or her weight on both feet. Then, in this position, three photos are taken in three numbers using the digital camera. Then, the digital photos are transferred to the computer and are analyzed using the USC software application (developed by the researcher) (Figure 1).

### Exercise Plan

The prescribed adjustment plan was designed for 12 wk, three sessions per each week, and each session lasting from 45 to 60 min. The exercises in the initial sessions had lower intensity, repetition, and duration. In later sessions, the exercises will grow more intense based on the capabilities of the participants and the exercises would become harder to follow the workout principles. The intensity of the exercises for stretching exercises was up to the pain threshold and for strength training, the exercises would continue until the individual was tired. All the exercises were performed based on workout principles, their intensity and its gradual increase, duration, the principle of increasing the load, and the movement pattern of engaging in exercise^2^ (Table 2).

### Ergonomic Training Intervention

**Figure 2:**
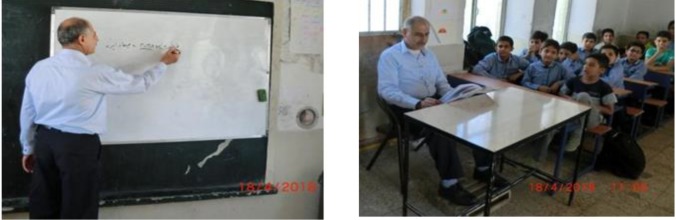
Ergonomic intervention

These training interventions were selected based on the protective and health directives of the Ministry of Labor considering the bodily conditions, muscular strength, and body movements in a way that unnecessary stress or too much stress on muscles, joints, ligaments, and the respiratory and cardiovascular systems were avoided. The interventions were provided for the participants in the form of the ergonomic pamphlet for carrying loads^17^. Some of the ergonomic training interventions used in the current study are as follows (Figure 2).

**Table 2:** Upper cross syndrome exercise

**Exercise: Self-Myofascial Release**	**Sets**		**Duration**		**Notes**
**Exercise (1-4** ^th^ **Week) - Inhibit**			
Latissimus Dorsi	1-3		30 sec		Thera cane & foam roll
Thoracic Spine	1-3		30 sec		Thera cane
Upper Trapezuse	1-3		30 sec		Thera cane
Sternocleidomastoid	1-3		30 sec		Thera cane
Levator Scapula	1-3		30 sec		Thera cane
**Exercise (5-8th Week) - Inhibit**	**Sets**		**Duration**		**Notes**
Latissimus Dorsi	1		30 sec		Thera cane & foam roll
Thoracic Spine	1		30 sec		Thera cane
Upper Trapezuse	1		30 sec		Thera cane
Sternocleidomastoid	1		30 sec		Thera cane
Levator Scapula	1		30 sec		Thera cane
**Lengthen**	**Sets**		**Duration**		**Notes**
Sternocleidomastoid Stretch	1-3		30 sec		Stretching
Levator Scapulae Stretch	1-3		30 sec		Stretching
Upper Trapezius Stretch	1-3		30 sec		Stretching
Ball Latissimus Dorsi Stretch	1-3		30 sec		Using ball
Standing Pectoral Stretch	1-3		30 sec		Stretching
**Exercise (9-12th Week) - Lengthen**	**Sets**		**Duration**		**Notes**
Sternocleidomastoid Stretch	1-2		30 sec		Stretching
Levator Scapulae Stretch	1-2		30 sec		Stretching
Upper Trapezius Stretch	1-2		30 sec		Stretching
Ball Latissimus Dorsi Stretch	1-2		30 sec		Using ball
Standing Pectoral Stretch	1-2		30 sec		Stretching
**Isolated Strengthening**	**Sets**	**Reps**	**Tempo**	**Rest**	**Notes**
Quadruped Ball Chin Tucks	1-2	10-15	4/2/2	0	Deep Cervical Flexors
Resisted Cervical Posterior Translation (chin tucks)	1-2	10-15	4/2/2	0	Cervical-Thoracic Extensors
Floor Prone Scaption	1-2	10-15	4/2/2	0	Lower Trapezius
Quadruped Ball Chin Tucks	1-2	10-15	4/2/2	0	Deep Cervical Flexors
Resisted Cervical Posterior Translation (chin tucks)	1-2	10-15	4/2/2	0	Cervical-Thoracic Extensors
Floor Prone Scaption	1-2	10-15	4/2/2	0	Lower Trapezius
Ball Combo I	1-2	10-15	4/2/2	0	Isolated Strengthening
**Integrated dynamic movement**	**Sets**	**Reps**	**Tempo**	**Rest**	**Notes**
Ball Combo I w/Cervical Retraction	1-2	10-15	Slow	30 sec	Using ball
Squat to Row	1-2	10-15	Slow	30 sec	Integration
Single-Leg Romanian Deadlift	1-2	10-15	Slow	30 sec	Integration
Standing 1-Arm Cable Chest Press	1-2	10-15	Slow	30 sec	Integration

1. Avoiding bending forward on the desktop and leaning on the front portion of the desk (to maintain the S-shaped structure of the spine);
2. Support was provided for the lower portion of the back;
3. The natural posture of the body in the neck and back areas was maintained and bending forward, backward, and to the sides was avoided;
4. Support was used for the feet;
5. Staying in a fixed position for a long time was avoided;
6. When teaching and writing on the whiteboard, the hand was kept in a place where the shoulders were less stressed (not very high and not very low);
7. Checking homework behind the students’ desks was avoided.

After the initiation of the study, the control group was asked to return to the research location after 12 wk and on specific dates for the post-test and they were asked to avoid any physical activity during this 12 wk and just perform their everyday activities and job activities.

### Statistical analysis

Demographic characteristics of the two groups were presented using descriptive statistics. Analysis of pairwise *t* -test and differential independent *t* -test were conducted to compare the effectiveness of NASM exercises with ergonomic intervention and routine activity on the study outcomes. The independent variable was the type of exercise (NASM exercises with ergonomic intervention and routine activity) and the dependent variables (forward head angle, rounded shoulders angle, hyperkyphosis angle) consisted of the study outcomes recorded after the exercise protocols were completed. Cohen's standard indicated effect sizes of each variable. Statistical analyses were carried out using SPSS ver. 23 software (Chicago, IL, USA). The level of significance was set at 0.05 for all tests.

## Results

Table 3 presents the descriptive information related to the measured variables in the pre-test and post-test for both groups.

**Table 3:** Effect of NASM exercise with ergonomic intervention on groups

**Group**	**Experimental**				**Control**				***P *** **value**
	**Pre**		**Post**		**Pre**		**Post**		
	**Mean**	**SD**	**Mean**	**SD**	**Mean**	**SD**	**Mean**	**SD**	
Forward head	47.08	2.79	39.90	4.83	45.50	2.09	45.29	1.92	0.001
Forward shoulder	55.43	4.213	47.85	4.86	57.59	3.86	58.10	3.34	0.000
Kyphosis	44.76	1.94	41.15	2.23	43.71	2.38	44.20	2.71	0.003

**Figure 3:**
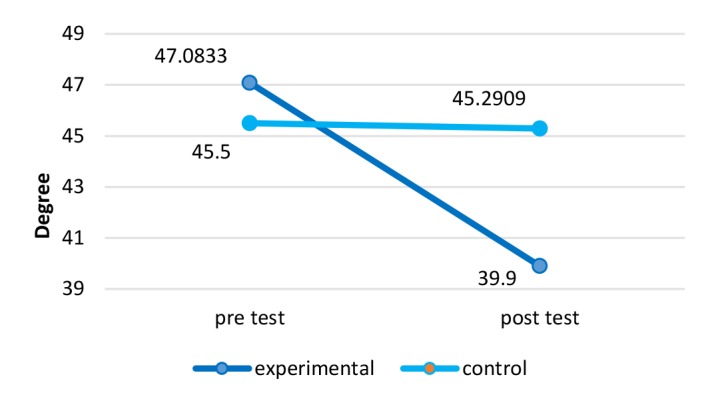
Forward head angel

**Head Forward Angle:**The results of head forward angles are demonstrated in Figure 3. There was a significant difference in the angle of forward head before and after 12 wk of NASM exercises with ergonomic intervention (*P*=0.001) in a way that the applied method had a 90% positive impact on reducing the intensity of this complication (Effect size=1.34).

**Figure 4:**
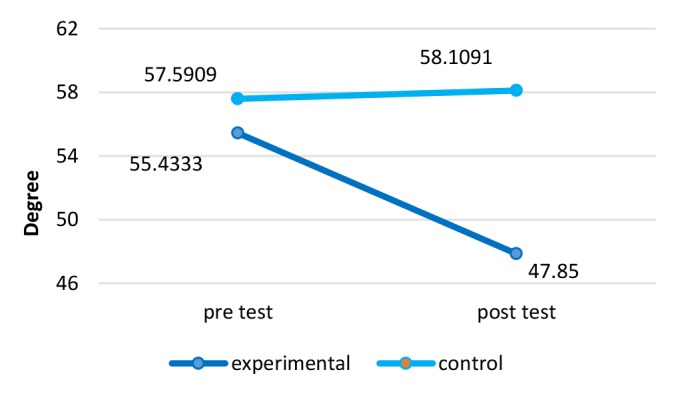
Rounded shoulders angel

**Rounded shoulders Angle:**The results rounded shoulder angles are demonstrated in Figure 4. There was a significant difference in the angle of rounded shoulder before and after 12 wk of NASM exercises with ergonomic intervention (*P*=0.000) in a way that the applied method had an 88% positive impact on reducing the intensity of this complication (Effect size= 1.28).

**Figure 5:**
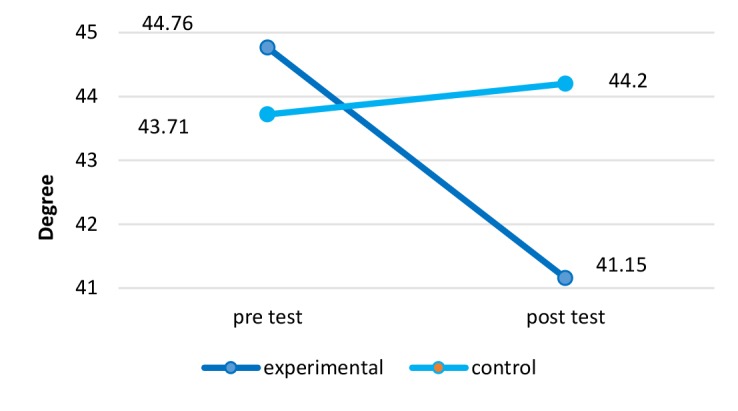
Kyphosis angel

**Kyphosis Angle:**The results of kyphosis angle are demonstrated in Figure 5.There was a significant difference in the angle of kyphosis before and after 12 wk of NASM exercises with ergonomic intervention (*P*=0.003) in a way that the applied method had a 90% positive impact on reducing the intensity of this complication (Effect size= 1.30).

## Discussion

In UCS, the shortening of upper trapezius muscles and scapula lifting muscles in the dorsal area is accompanied by the shortening of small and large chest muscles as well as the weakening of deep neck flexors and middle and lower trapezius. This asymmetric pattern leads to the interference in the functioning of joints, particularly the atlanto-occipital joint, C4-C5 segment, Cervical-Dorsal Joint, and the glenohumeral joint^18^. Moreover, scholars suggest that weakening of retraction muscles such as trapezius and equilateral muscles (rhomboid muscles) increase the scapula abduction. According to the general principles of corrective and therapeutic exercises, strengthening the weakened muscles leads to biomechanical movement and appropriate positions for abnormal areas. NASM exercises and ergonomic interventions in the current study were focused on the muscles involved in this complication, and they were designed and applied based on the chain reaction theory of Janda and the gear mechanism of Bruger^19^. The selected exercises had been designed in a way that they could affect all three abnormalities (forward head, rounded shoulders, and kyphosis) simultaneously and the individual would perform the exercises in an active and dynamic fashion. Probably, introducing such movements in the exercise protocol has contributed to the improvement of these abnormalities. Therefore, this finding is in line with the results of some studies ^4, 5, 14, 20, 21^.

These are in contrast to Kendal theory who believes that exercises must be performed in a localized and isolated manner^1^. Kendal’s view for improving postural abnormalities is based on stretching the shortened muscles and strengthening the weakened muscles in the involved location. It is rarely the case that a part of the body is afflicted with a deformity on its own; rather, deformities in one part of the spine will affect other parts. The interdependencies of the effects of abnormalities and complications in various parts of the body on each other must be considered during therapy ^1^.Despite the reduction in the angle of kyphosis for participants after attending the corrective exercise program, these exercises do not have a satisfactory effectiveness ^19^. This issue is due to focusing on localized corrective exercises and neglecting other abnormalities and complications related to kyphosis.

Moreover, the exercises in this study had a 90% positive impact on reducing forward head angle, an 88% positive impact on reducing rounded shoulder angle, and a 90% positive impact on reducing the kyphosis angle. Therefore, considering the significant decrease in forward head, forward shoulder, and kyphosis angles of the participants in the experimental group in this study, the NASM corrective exercise program accompanied by ergonomic intervention have satisfactory effectiveness. The effectiveness of NASM exercises was also supported by other studies ^5, 20, 22^.

The difference between NASM exercises and other exercises is the inhibition stage. This stage focuses on inhibition or relaxation of hyperactive muscles, probably created due to repetitive movements or for protecting the damaged area or compensating for weaker muscles^23^. In muscles shortened during deformity, myofascial adhesions and trigger points are created^24^. The objective of inhibitive techniques is to release tension or reduce the hyperactivity of neural-muscular- fascial texture in the body. Using myofascial release techniques creates an inhibitive response in the muscle spindles and the release of stiffed and shortened muscles^2^. Using foam rolls and tracking are good methods for this stage and in this study, the myofascial release exercises were performed by the individuals themselves using foam rolls and tracking, which can be one of the reasons behind the effectiveness of this exercise protocol.

The highlighted characteristic of the exercise protocol in the current study is ergonomic interventions. Nowadays, much attention is given to ergonomics in various occupations and working environments. The effects of occupational factors in the appearance of postural abnormalities as well as joint pains and musculoskeletal pains. Techniques for preventing such complications involves educating people on the proper mechanics of the body during occupational activities and changes in the mechanical pressure of the occupation based on the ergonomic process^8^.

Upper cross syndrome pattern is often experienced among people who sit for long periods or people who exert frequent overload patterns on their upper body organs. Bruger, the Swedish neurologist, defines gear process for the spine as the fact that the sedentary posture will lead to the posterior rotation of the pelvis, which reduces the natural lordosis of the lumbar spine, the natural kyphosis of the lumbar spine with the gear movement is intensified in a clockwise direction, and it ultimately creates a gear movement in anticlockwise direction in the neck vertebrae. The terminal gear leads to forward head posture in weak postures^2^. On the other hand, while sitting behind a desk, teaching and writing on the whiteboard, checking the students’ homework on their desktops, and teaching and supervising the class while standing, teachers use improper postures, which lead to musculoskeletal deformities and complications^25^. For instance, increased dorsal kyphosis during work is closely related to the movement of the head and the cervical spine^26^. Therefore, by providing ergonomic instructions in the working environment, and correcting the posture in working situations for teachers, the study lead to the high effectiveness of the exercises.

The limitations of this study were the population sex, which only men included in this study, as because of nature of this study it was necessary to evaluate without clothing. The implication of this study is importance of using this exercise programme for the teachers who suffer from USC.

## Conclusion

NASM exercises along with ergonomic interventions could be an effective rehabilitation program for reducing forward head, forward shoulder, and kyphosis angles. Moreover, the UCS software application could be used as an accurate instrument for measuring the extent of the upper cross syndrome.

## Acknowledgements

The authors thank the teachers who participated in this study.

## Conflict of interest

The author(s) declared no potential conflicts of interest with respect to the research, authorship, and/or publication of this article.

## Funding

The present study was financially supported by University of Isfahan.

## Highlights

- The use of UCS software for measuring the extent of upper cross syndrome.
- Exercises with ergonomic training intervention reduce the forward head angle.
- Exercises with ergonomic training intervention reduce the rounded shoulders angle.
- Exercises with ergonomic training intervention reduce the kyphosis angles.
